# A systematic review of public views on the reintegration of men convicted of a sexual offense into the community

**DOI:** 10.1177/15248380251325816

**Published:** 2025-03-18

**Authors:** Emma Tuschick, Shiri Portnoy, Nikki Carthy, Laura Gair, Simon Hackett, Nadia Wager

**Affiliations:** 1 Teesside University, Middlesbrough, UK; 2 Cumbria, Northumberland, Tyne & Wear NHS Foundation Trust, UK; 3 Newcastle University, UK

**Keywords:** offenders/perpetrators, sexual assault, sexual assault, sexual abuse, child maltreatment, prevention, sexual assault

## Abstract

This systematic review explores public views on the reintegration of men convicted of sexual offenses into the community. A search of eight databases produced 8,621 potential sources, and after screening 12 studies were included in the review. Papers were included if they used qualitative methods about the public’s views, attitudes, opinions, and/or perceptions on the reintegration of adult male sexual offenders from prison or secure care. The papers were then critically appraised and thematically synthesized. The findings highlighted four key themes: supervision, discrimination, livelihood, and interventions. Public perspectives of men convicted of a sexual offense reintegrating into the community were generally negative, fueled by media portrayals and misconceptions about the risk of reoffending. These views lead to support for stringent monitoring and restrictions, often at the expense of rehabilitation efforts. While some members of the public advocated for supportive reintegration programs, others emphasized punitive measures and expressed distrust in the effectiveness of rehabilitation. The review also highlights the significant impact of public stigma on the daily lives of offenders, particularly in relation to housing and community acceptance. The implications for future research, policy, and practice, including public education campaigns, community involvement, and enhanced support systems for reintegration, are discussed.

## Background

Sexual offenses represent a significant social issue, both in terms of their prevalence and the lasting impacts they have on individuals and communities. In the United States alone, it is estimated that over 433,000 rapes and sexual assaults occur each year, with similar concerns reflected in many other nations (Charlie Health, 2024). Society at large grapples with the tension between the need to protect potential future victim-survivors, therefore having a strong desire for the punishment of men convicted of sexual offenses (MCoSO; including the death penalty), and the principles of rehabilitation and reintegration (Mancini, 2018). This tension is reflected in the social responses to MCoSO, which often focus on punitive measures rather than rehabilitation or reintegration, driven in large part by negative public attitudes.

Multiple studies across various countries, including the United States, the United Kingdom, Norway, and Australia, consistently show that the public holds stigmatizing views toward MCoSO, perceiving them as a persistent threat to community safety (Bartels et al., 2020; Sandbukt, 2021; Shi et al., 2022; Tovey et al., 2022). These attitudes, largely influenced by sensationalized media portrayals and political rhetoric, further compound the challenges faced by MCoSO and shape restrictive criminal justice policies (Harper et al., 2018; Sandbukt, 2019). However, little attention has been paid to how these punitive attitudes might undermine efforts toward rehabilitation and reintegration, highlighting the need for a comprehensive review of the studies that explore these dynamics.

Attitudes toward MCoSO can vary depending on the specific case and the circumstances surrounding the offense. For example, those who commit a sexual offense against a child are often the most disdained group by the public (Jahnke & Hoyer, 2013; Jung et al., 2012). Even without scientific evidence, the public perceives the “sex offender register” to be effective and fair and often holds on to “sex offender myths,” such as most offenders do not know their victims, the likelihood of sexual recidivism is high, all offenders are the same, and that offenders cannot be rehabilitated (Olga Sánchez et al., 2024; Zatkin et al., 2022).

Demographically, women tend to be more punitive and hold negative thoughts toward MCoSO than men (Willis et al., 2013). Other common demographics of individuals who were more negative toward MCoSO included those with lower levels of education (Willis et al., 2013), white, and those in nonprofessional roles (Winnick, 2008). Additionally, the public has been found to have overwhelming distrust in the government being able to manage MCoSO effectively and believe they themselves should be more involved in this (McCartan, 2013). The public’s views on reintegration are not rigid; they encompass a diverse range of perspectives, from those advocating for stricter monitoring and lifetime penalties to those who emphasize the potential for redemption and reintegration (Kras, 2022; Tuschick et al., 2024). Bridging these opposing viewpoints is challenging but essential to develop policies and practices that reflect the values and concerns of the communities affected by sexual offenses.

There is an ongoing debate about the effectiveness of current policies worldwide for addressing sexual offending and the potential for rehabilitation for offenders. The negative stereotypes and stigmatization of MCoSO by the public influence current and often harsh restrictions placed upon them in many different countries (King & Roberts, 2017; Sparks, 2021). Registration policies differ significantly by country. For example, in the United States, lifetime registration is common and widely supported by public sentiment, while countries such as the UK and Canada implement more nuanced policies that provide for periodic review and potential removal from registries based on individual rehabilitation progress (T. Thomas, 2012; Zgoba & Cowan, 2019). These differences highlight how legal frameworks are shaped by cultural and political contexts, with some systems focusing more on rehabilitation and reintegration than others.

Research shows that lifetime registration can have harmful psychological effects, including anxiety, depression, and feelings of hopelessness, as it permanently brands individuals as offenders, even after rehabilitation efforts (Vess et al., 2013). In addition, while public sex offender registries have strong public support, studies indicate they have limited effectiveness in reducing recidivism and fail to significantly alleviate community fears (Napier et al., 2018). Moreover, lifetime registration and restrictive residency laws can hinder access to social support and rehabilitation services, which are crucial for reducing recidivism (Tewksbury, 2007).

Societal treatment of MCoSO often results in negative consequences, such as social isolation, barriers to employment, and restricted access to housing. These challenges are exacerbated by the widespread use of public sex offender registries, with some individuals subject to lifetime registration. Studies indicate that these harsh measures may increase the likelihood of reoffending, as they foster conditions of marginalization and desperation rather than supporting reintegration (Levenson & Cotter, 2005). Thus, these policies may inadvertently contribute to further harm, highlighting the need for more balanced approaches that emphasize rehabilitation and community reintegration (Bailey & Sample, 2016).

Attitudes and knowledge about sex crimes are constantly evolving and are influenced by many factors, such as cultural, social, legal, and political contexts (Harper et al., 2018; Rosselli & Jeglic, 2017). In an evolving societal landscape, education and awareness campaigns, alongside rigorous research, are crucial for improving public attitudes and knowledge regarding sexual assault. Such efforts can significantly enhance the support provided to victim-survivors and lead to more informed and effective responses to sexual offending (McCartan et al., 2021). By challenging misconceptions and fostering a more nuanced understanding, these initiatives can contribute to a safer and more just society.

Currently, there is a compelling argument that the societal treatment of MCoSO may inadvertently contribute to further harm and potential reoffending. This highlights the urgent need for increased awareness to reduce future sexual crimes (Lawrence & Willis, 2021). The literature clearly demonstrates the necessity of thoroughly investigating and understanding people’s views and opinions and the prevalence of myths surrounding the reintegration of MCoSO. Given the significant influence the public exerts over legal restrictions, sentencing, and the overall management of MCoSO by the Criminal Justice System (CJS; Cochran et al., 2020) it is crucial to address these perceptions.

This review is essential to examine not only the societal attitudes that fuel harsh policies but also the evidence that questions the effectiveness of such approaches. The public’s misconceptions, such as the belief that sexual offenders are unlikely to be rehabilitated, necessitate a clearer understanding of how these attitudes influence legislation and whether current policies serve to reduce recidivism or, conversely, exacerbate the problem by marginalizing offenders (Levenson & Cotter, 2005). Therefore, the aim of this systematic review is to comprehensively and qualitatively explore the public’s views on the reintegration of MCoSO from prison or secure care into the community. This exploration is essential for developing informed and balanced policies that can effectively support both public safety and the rehabilitation of offenders.

In light of this focus on public perceptions, qualitative studies are particularly well-suited for this review, as they delve into the nuanced experiences and attitudes of individuals, capturing the complexities of societal beliefs surrounding MCoSO (Agius, 2013). While quantitative studies provide valuable statistical insights, they often lack the depth needed to understand the underlying motivations and emotions that drive public opinion (Agius, 2013). Thus, the choice not to include quantitative studies stems from the recognition that they may not exist in this specific context, but more importantly, because qualitative evaluations offer a richer, more comprehensive understanding of the social dynamics at play. This systematic review makes a substantial contribution to the field by addressing a critical gap in the literature surrounding public views toward MCoSO. While previous reviews have primarily focused on quantitative analyses (Zatkin et al., 2022), or overlooked this specific area entirely, this study distinguishes itself by delving into qualitative research, offering a nuanced exploration of public perceptions and attitudes toward MCoSO.

## Methods

The protocol for this systematic review was registered with PROSPERO under the identifier CRD42023453446, and no changes have been made since its registration. The review adheres to the 2020 PRISMA reporting guidelines (Shamseer et al., 2014).

### Search Strategy

Database searches were performed using the Population/Exposure/Outcomes framework, which was deemed most appropriate for the qualitative synthesis of this review (Mattisson, 2023). The Population referred to the general public, Exposure focused on the reintegration of men convicted of sexual offenses, and Outcome encompassed views, opinions, perceptions, and attitudes. A sample search strategy is provided in Supplemental File 1. All searches were carried out in September 2023, and to ensure comprehensive coverage, searches were specifically aimed at identifying key studies that addressed the research questions to enhance the search’s sensitivity. In addition, the papers’ bibliographies were screened for additional papers.

A comprehensive search was conducted across eight electronic databases: MEDLINE, PsychINFO, Web of Science, Criminal Justice Abstracts, ProQuest, PubMed, Psychology and Behavioral Sciences Collection, and CINAHL. In addition, two gray literature sources, Google Scholar and MEDNAR, were explored, with the first 10 pages (100 results) retrieved from each to capture the most pertinent information (Haddaway et al., 2015). The primary databases were selected to ensure comprehensive coverage and maximize the retrieval of relevant papers. To confirm the adequacy of this approach, a gold standard paper was initially used to verify that all pertinent studies were successfully identified, eliminating the need for additional database searches. All retrieved data were then imported into Rayyan, where duplicates were eliminated before the screening process began.

### Inclusion and Exclusion Criteria

Studies were eligible for inclusion if they employed qualitative research methods, or mixed methods (providing the qualitative data could be extracted separately) to explore the public’s views, attitudes, opinions, and/or perceptions regarding the reintegration of adult male sexual offenders from prison or secure care, that were published in the English language. The restriction to English-language studies was necessary due to resource constraints, such as the availability of translation services and the potential for misinterpretation in non-native languages. While this limitation reduces the inclusion of diverse perspectives from non-English-speaking regions, it was important to ensure the accuracy and consistency of data analysis within the scope of the review. Journal empirical papers were also included if they contained perspectives from sexual offenders themselves, provided that data from the public could be extracted. In addition, studies that did not primarily focus on reintegration but included relevant data on the topic were also considered, provided that the reintegration-related data was clearly extractable and formed a meaningful part of the study. Eligible sources were explicitly limited to peer-reviewed journal empirical papers, book chapters, and books. Reviews, commentaries, opinion pieces, and conference abstracts were excluded, as were studies lacking sufficient qualitative data on public attitudes toward reintegration. There were no restrictions on the country of origin or publication year of the papers.

### Study Selection and Data Management

After removing duplicates, one reviewer (ET) examined all titles and abstracts based on the inclusion and exclusion criteria. A second reviewer (NW) then independently reviewed 20% of the papers chosen at random. The two reviewers achieved a 98.9% agreement rate, which corresponds to a substantial agreement level when expressed as a Kappa statistic, and no discrepancies were referred to a third reviewer. Papers deemed potentially relevant proceeded to a full-text screening. All full papers were retrieved and stored on Microsoft Teams for review. Reviewer ET evaluated all full papers, while reviewers S.P. and L.G. independently double-screened 30% of the papers, achieving a 73.3% agreement rate, indicative of moderate agreement according to the Kappa statistic. To address this moderate agreement, the reviewers discussed any differences thoroughly and revisited the assessment criteria to resolve ambiguities and ensure a consistent understanding. These steps helped mitigate the moderate agreement rate and ensured a thorough and reliable quality assessment of the included papers.

### Data Extraction

A Microsoft Excel spreadsheet was developed for the data extraction which captured: the authors, year of publication, country of study, the aim of the research, study design, methods, setting/location of research, sample size, sex offense type, participant demographics, sample positive results, sample negative results, recommendations, and conclusions.

### Assessment of Quality

The quality of the included papers was assessed using the CASP tool for qualitative studies, a widely used checklist designed to evaluate the rigor, credibility, and relevance of qualitative research (Singh, 2013). Endorsed by Cochrane and the World Health Organization for qualitative evidence synthesis, the CASP tool is recognized for its effectiveness in evaluating research transparency and reporting standards (Long et al., 2020). This systematic review’s aim was not to assess the quality of the papers; therefore, the CASP tool was used, which suggests not to use a scoring system. One reviewer (ET) conducted the quality assessments for all papers, while a second reviewer (LG) independently reviewed 20% of the papers. The two reviewers achieved a 100% agreement rate, reflecting perfect agreement according to the Kappa statistic. No papers were excluded at this stage.

### Synthesis

To synthesize the extracted data, an inductive thematic synthesis approach was employed, with data line-by-line coded using NVivo 12 software, London, United Kingdom. Thematic synthesis was selected for its ability to provide a thorough understanding of qualitative evidence by organizing it into common themes, patterns, and variations (J. Thomas & Harden, 2008). This method’s flexibility ensures that all relevant insights from the included papers are captured. Furthermore, the structured approach of thematic synthesis, involving data coding, categorization, and theme development, promotes consistency, and transparency and enhances the credibility of the review’s findings (Nowell et al., 2017). Initial drafts of the synthesis were prepared by ET and subsequently reviewed by the broader study team (NW, NC, SP, LG, SH), who reached consensus on the themes and sub-themes, thus strengthening the trustworthiness and rigor of the synthesis (Maher et al., 2018).

## Results

**Figure 1.**
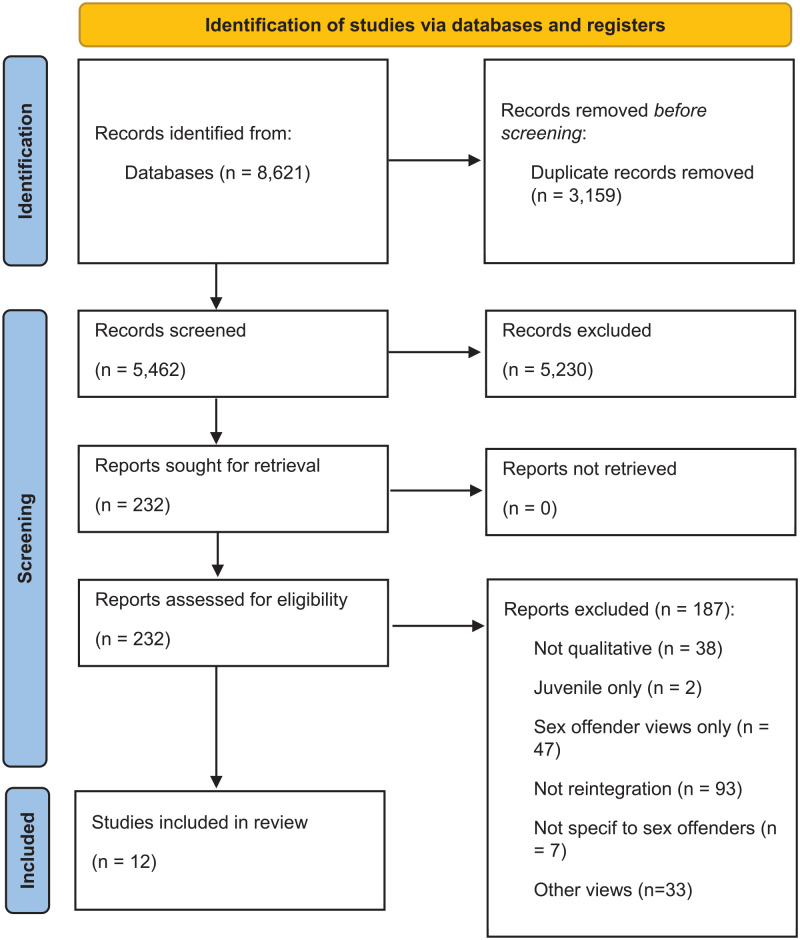
PRISMA. *Source*. Page et al. (2021).

The initial search identified 8,621 records. After removing duplicates (3,159 removed) and filtering titles and abstracts (5,230 removed), 232 full-text papers were evaluated. Ultimately, 12 papers met the inclusion criteria and were included in the review (see Figure 1). These articles were published between 2004 and 2020.

### Study Characteristics

The details of the 12 qualitative studies (Beshears, 2017; Boone & Henk van, 2016; Brown et al., 2008; Chen, 2020; Lowe et al., 2019; Lowe & Willis, 2019, 2020; McCartan, 2013; Richards & McCartan, 2018; Taylor, 2017; Thakker, 2012; Zevitz, 2004) are summarized in Table 1. These papers explored public perspectives on the reintegration of adult male sexual offenders, including views from academics, the general public, the media, and neighbors. The studies involved a total of 4,790 participants and covered mixed sexual offenses. Research came from various countries: three studies were conducted in the United Kingdom, four were conducted in New Zealand, two each in the United States and Australia, and one from the Netherlands.

**Table 1.** Study Characteristics.

Author, Year, Location	Methods	Total sample size (*N*)	Viewpoint
Beshears (2017), USA	Interviews	20	Neighbors
Boone and van de Bunt (2016), The Netherlands	Interviews	48	The public
Brown et al. (2008), UK	Focus groups and questionnaires	979	The public
Chen (2020), China	Data analysis	2261	The public
Lowe et al. (2017), New Zealand	Interviews	18	The public
Lowe and Willis (2018), New Zealand	Interviews	18	The public
Lowe and Willis, (2019), New Zealand	Mixed methods survey	310	The public
McCartan (2013), UK	Focus groups	35	The public
Richards and McCartan (2017), Australia	Data analysis	768	The public
Taylor, 2017, Australia	Mixed methods survey	162	The public
Thakker (2010), New Zealand	Focus groups	23	The public
Zevitz (2004), USA	Interviews and surveys	128	Neighbors

### Thematic Results

The thematic analysis revealed four main themes regarding the reintegration of MCoSO: supervision (encompassing registry issues, restrictions, parole and probation conditions, policies, and monitoring), discrimination (covering labeling, stigma, vigilantism, acceptance of myths, and stereotyping), livelihood (addressing challenges related to housing), and interventions (including rehabilitation, support groups, and therapy).

#### Theme 1: Supervision

Eight papers looked at the “sex offender” registry, restrictions, parole, and probation conditions, policies concerning this area, and monitoring.

There was a recurring theme of the need for monitoring and supervision of McoSO among the public, both in the community and upon release, which appeared more prevalent for pedophiles than any other sexual offense. This included suggestions for day-to-day monitoring of activities and stringent parole conditions.

The day-to-day activity of certain sex offenders should be monitored to the extent that, for the most part, their routine is known. From this, any potential situations could possibly be avoided. Avoiding danger to the community should be paramount. (Brown et al., 2008)

However, there was tension between prioritizing community safety and supporting the rehabilitation and reintegration of MCoSO. Some expressed the belief that offenders should be closely monitored for the safety of the community, while others advocated for providing support to help offenders reintegrate into society.

But I suspect the most effective monitoring is to give the offender the space to allow them to act responsibly and the support to enable them to overcome their problems and learn to take responsibility for their actions. But I recognize that there is (as yet) no cure for sexual deviance and so a strategy of monitoring aimed at risk reduction would seem the most appropriate. (Brown et al., 2008)

Indeed, the papers highlighted the stigma associated with MCoSO and the impact it has on their reintegration into society. There were concerns about the demonization of offenders and the potential for vigilante action fueled by public outrage.

“Yep! That’s the spirit. We despise the ex-cons and they need to know it. The low lives should live in the dirt for life!”; “Sex offender registration will fuel the fear of crime. It will reduce people’s sense of security and entrench the distrust and isolation between individuals. Eventually everybody will be seen as a potential threat. This impact can do more harm to the society than sexual offences per se.” (Chen, 2020)

There were mixed opinions on the effectiveness of “sex offender” registration and disclosure schemes, which involve allowing the public or certain individuals access to information about “registered sex offenders” in their area. Some believed they were necessary for community safety, while others questioned their efficacy and raised concerns about potential negative consequences, such as stigmatization and a false sense of security.

It stigmatizes sex offenders in a way that is a sad indictment on our society. Perhaps a compulsory Auschwitz-style tattoo will help identify more readily sex offenders as they go about trying to re-integrate into society. (Taylor, 2017)

Despite concerns about monitoring and supervision, there was recognition of the importance of providing support and rehabilitation opportunities for offenders. Some emphasized the need for offender treatment programs and support systems to address the underlying issues contributing to offending behavior.

Exactly, finding the right sort of support system for them, not just dumping them somewhere there they think, ah there is a bunch of people, but having the right people that are going to do the right things for them and care about them enough to put these things in action and support them all the way through. (Thakker, 2012)

The balance between community safety and the reintegration of MCoSO is a delicate one, fraught with tension and differing opinions. While stringent monitoring and supervision were seen as necessary by some to protect the public, others advocated for a more supportive approach that facilitates rehabilitation and reintegration. Geographical and cultural factors played a significant role in shaping these attitudes. In countries such as the United States, public opinion leaned more heavily toward community safety, whereas in the UK and New Zealand, there was greater emphasis on offender rehabilitation and support, illustrating the regional differences in how supervision policies were perceived.

#### Theme 2: Discrimination

Eight papers looked at labeling, stigma, vigilantism, myth acceptance, and stereotyping of MCoSO.

Stigma and public perception regarding MCoSO, especially those who have victimized children, were prevalent issues within the public. The stigma surrounding this group often led to overestimation of the risks they posed, perpetuating negative stereotypes fueled by media reporting. Misconceptions about the nature of sexual offenses, the likelihood of reoffending, and the differentiation between various types of offenders further contributed to this stigma. Community responses to MCoSO represented a broad spectrum, ranging from supportive to hostile attitudes. Initiatives such as Circles of Support and Accountability (CoSA), which are community-based initiatives led by experienced practitioners and supported by volunteers and designed to assist individuals with a history of sexual offenses as they reintegrate into society, exemplified community-driven efforts aimed at fostering reintegration and lowering recidivism rates by offering support and fostering accountability for offenders.

I mean it’s something that requires help and treatment, right? As opposed to any kind of vitriolic ‘let’s castrate all the pedophiles, those horrible monsters. I really can’t stand that kind of stuff and interestingly, I think it’s the kind of public reaction and vitriol against child sex offending that makes me think CoSA is even more valuable and important, because there aren’t that many groups in society that are treated . . . just universally ostracized and totally hated on. (Lowe et al., 2019)

Within communities, there existed a diversity of perspectives, with some advocating for more empathetic and rehabilitative approaches toward MCoSO, while others prioritize punitive measures and emphasize support for victim-survivors. The influence of misinformation was especially strong in the US and the UK, where public demands for punitive measures were more pronounced.

I think having actually worked in the prison system made a big difference to me—made me realize that prisoners are people. I think until then I had had the attitude, which I think a lot of us have, ah you know, ‘he’s committed a crime, lock him up, throw away the key. (Lowe & Willis, 2019)

I cannot relate in any way, shape or form to a sex offender or a child sex offender. I therefore could not possibly have anything positive to contribute by volunteering with them. (Lowe & Willis, 2020)

Opinions suggested that the media wielded substantial influence in shaping public perceptions of MCoSO, frequently sensationalizing cases and thereby amplifying fear and insecurity within communities. This portrayal often contributed to misconceptions and exacerbated the stigma surrounding these men. This also led to discussions surrounding vigilantism against MCoSO which highlighted concerns about the potential risks faced by offenders within the community.

The argument is that these people will become the target of vigilante action, and the occurrence of this is rare, in the balance, compared to the cases where children are abused”; “I’d say the community would not care what happened to him, now come on, I think it depends on who got to him ﬁrst, the police or the community, it would just depend on how lucky he was. (McCartan et al., 2021)

These varied responses emphasized the complexity of addressing the issue of MCoSO within communities and the importance of considering a range of approaches to effectively support both offenders and those impacted by their actions. Individual perspectives on MCoSO and rehabilitation efforts were found to be heavily influenced by personal experiences and beliefs.

#### Theme 3: Livelihood

Nine papers looked at the public perceptions mainly surrounding housing of MCoSO and the effects this can have on the community.

Community concerns and fears surrounding the presence of MCoSO in neighborhoods manifested through varying degrees of apprehension, with a particular emphasis on the safety of children. Residents commonly harbored fears stemming from the unknown and apprehensions regarding personal and familial safety.

I felt really sad, why were they put in a residential area? They should be out in the country; they should be isolated being sex offenders. They should not be in residential areas with schools and children. (McCartan et al., 2021)

Many expressed discomfort, anxiety, or feelings of threat associated with the proximity of MCoSO. To counteract perceived risks, measures such as heightened vigilance, secured doors, and keeping blinds closed were adopted as precautionary measures within the community. These sentiments highlighted the significance of addressing community anxieties and implementing strategies to foster a sense of security and well-being among residents.

I think that is what everybody’s concern is, you know. It’s fear of the unknown. What are they going to do? Umm, so it’s, it’s the way you would in your head to get them back into society rehabilitated them but in your heart that fear of something happening. (Thakker, 2012)

The desire for information and notification regarding the past offenses of MCoSO residing nearby was a common attitude among many participants, underlining the significance of transparency and community awareness. Residents emphasized the importance of being informed about the criminal history of offenders in their vicinity. Generally, notification systems implemented by authorities, such as those managed by sheriff’s offices, were welcomed and appreciated by residents as they provided valuable insights and contributed to a greater sense of safety and security within the community.

I am glad that the sheriff's ofﬁce sends out notices. I didn’t realize that they would keep doing this every year, and I am glad because what if I had just moved in the house. (Beshears, 2017)

The presence of MCoSO in the neighborhood significantly impacted residents’ daily lives, social interactions, and overall sense of safety. Participants reported alterations in behavior, including avoiding specific areas or curtailing outdoor activities, stemming from apprehensions regarding the proximity of offenders; *“Now I won’t let my grandkids go play in the park up there unless I’m with them or somebody else I know is”* (Zevitz, 2004).

Moreover, the presence of MCoSO had repercussions on local businesses and property values, prompting some residents to contemplate relocation. These effects highlighted the impact that the presence of offenders had on the daily lives and well-being of residents within the community, prompting considerations for mitigating strategies and support mechanisms.

It’s just not fair that he should be placed here. It’s going to inhibit future people renting here, both business rentals and apartment rentals. Anybody knowing about this is going to think twice. I’m sure everybody around here feels the same as I do . . . It doesn’t feel safe knowing he’s here. (Zevitz, 2004)

However, community mobilization and support for MCoSO were evident in certain areas where residents believed in the significance of rehabilitation and providing individuals with second chances. Informal support networks, forgiveness initiatives, and community engagement with offenders were observed as efforts toward encouraging rehabilitation and integration; *“A convicted sex offender has to live somewhere”; “Everyone deserves a second chance”* (Boone & Henk van, 2016).

Additionally, neighborhood meetings and discussions facilitated by local authorities were viewed as beneficial avenues for addressing community concerns and promoting understanding between residents and offenders. These initiatives highlighted the importance of community involvement and compassion in facilitating the reintegration of offenders into society: “*It’s not just the community’s safety, it’s his safety. You keep him safe, you keep the community safe*” (Lowe et al., 2019).

#### Theme 4: Interventions

Five papers looked at interventions, rehabilitation, support groups, and therapy that were offered to MCoSO.

There was a clear divide between pessimistic views that believed MCoSO were unlikely to change or be rehabilitated, and more optimistic perspectives that advocated for rehabilitation efforts and support services that addressed underlying causes of offending behavior.

I believe that sex offenders have a character defect that will cause them to reoffend as soon as they think they can get away with it. The only sure way to control them is to keep them behind bars until they die. (Brown et al., 2008)

I have compassion toward people in prison, they are human beings capable of rehabilitation; their time in prison can be productive for them and our community. (Lowe & Willis, 2020).

Views on the effectiveness of treatment and rehabilitation programs also varied. While some expressed doubts about the efficacy of such programs, such as those overestimated re-offending rates (“*They are extremely likely to reoffend*” (Brown et al., 2008)), others believed in their importance and advocated for greater investment in rehabilitation efforts.

The penal system is altogether backwards if not done with the end-goal of rehabilitation. No effort should be spared in attempting rehabilitation of all persons who have found themselves outcast from society. You cannot simply ignore certain groups, the attempt is to reduce recurrence of all offending so to not volunteer to rehabilitate those who have done the most heinous crimes is only going to ensure that they’re the ones that are re-committed. (Lowe & Willis, 2020)

There was a recognition of the importance of community support in the rehabilitation and reintegration process. Participants highlighted the significance of relationships, support networks, and community connections in facilitating successful reintegration and reducing reoffending.

He is a good friend and I see that as being really important . . .a really important part of his reintegration into society is to have people that he can trust and rely on and feel comfortable with. (Lowe & Willis, 2019)

However, some participants expressed concerns about manipulation from MCoSO and the need for professional involvement.

I’m not disturbed by the nature of the offending but I think sex offenders have rehabilitative needs that untrained people like myself are unlikely to be able to help with. (Lowe & Willis, 2020)

## Discussion

General Discussion

The goal of this systematic review was to thoroughly examine the viewpoints of the public regarding the reintegration of MCoSO from prison or secure care into the community with four main themes being discussed: supervision, discrimination, livelihood, and interventions. These themes reflect the public’s diverse perspectives on MCoSO, highlighting a tension between community safety and the principles of rehabilitation and reintegration.

From the public’s viewpoint, rigorous monitoring and supervision of MCoSO, especially those convicted of pedophilia, were viewed as essential for community safety. The effectiveness of “sex offender” registration and disclosure schemes was debated by the public. While some viewed these measures as necessary for community safety, others questioned their efficacy and highlighted their potential negative consequences, such as stigmatization and a false sense of security. Research has consistently shown that overly punitive measures can be counterproductive, leading to recidivism rather than reducing it (Brooks & Jay, 2017; Lacey & Pickard, 2015; Maruna, 2007). Effective rehabilitation programs that address underlying psychological issues, provide social support, and facilitate gradual reintegration are crucial for reducing reoffending rates (Andrews & Bonta, 2014). The findings from this review demonstrate that such attitudes are not homogenous but vary across different public groups (such as neighbors and the media), as well as by the offender’s crime type and region.

Public attitudes toward MCoSO and their reintegration were generally found to be negative and stigmatizing, which is consistent with the wider literature, which shows these offenders are often viewed as perpetual dangers to the community (Klein et al., 2020; Socia et al., 2021). Moreover, the literature indicates that the public’s understanding of sexual offending is often limited, leading to misconceptions about the risk of reoffending and the effectiveness of rehabilitation (Mancini et al., 2010). The stigma and discrimination found in this review were often perpetuated by media reporting, which continuously sensationalized cases, leading to misconceptions about the nature of sexual offenses and the likelihood of reoffending. The media’s role in shaping public perceptions is critical. Sensationalized media coverage often leads to heightened fears and misconceptions about MCoSO, contributing to stigma and potentially inciting vigilantism (McCartan, 2013). This highlights the need for balanced and accurate reporting to prevent the spread of misinformation and to foster a more informed and rational public discourse on the issue (Rokach & Patel, 2021; Sandbukt, 2019).

The public’s reliance on the “sex offender register” and the persistence of myths surrounding sexual offending further compounded these negative attitudes, making reintegration challenging and often reinforcing stigma and hindering reintegration. While the public views registries as necessary for community safety, skepticism exists about their actual effectiveness in reducing recidivism. Instead, they often perpetuated misconceptions, such as the belief that all offenders are irredeemable and highly likely to reoffend, further marginalizing individuals and limiting their access to critical resources like housing and employment. These findings align with previous literature highlighting the impact of public stigma on reintegration efforts (Harper et al., 2018; King & Roberts, 2017). Interestingly, public attitudes can vary significantly based on the offender’s profile. For example, public perceptions tend to be more lenient toward high-profile individuals accused of sexual offenses, suggesting a bias influenced by social status (Valliere, 2022). This highlights the need for public education campaigns to address misconceptions and promote a balanced understanding of sexual offending and reintegration. Studies have shown that targeted public education initiatives can effectively shift attitudes, making the public more receptive to rehabilitative approaches (Willis et al., 2013). Reducing public stigma is crucial, as it can lead to social isolation and significant barriers to reintegration (Harper et al., 2018; King & Roberts, 2017; Levenson & Cotter, 2005).

In comparison to a recent systematic review looking at the barriers and facilitators to the reintegration of MCoSO from the offender’s point of view (Tuschick et al., 2024) with this review, both highlight the pervasive challenges in the reintegration of MCoSO, reflecting the complexities of societal attitudes and the need for balanced rehabilitation efforts. Public perspectives were also predominantly negative and shaped by media and political discourse, resulting in the demand for stringent restrictions and social ostracism. Encouraging community involvement in reintegration efforts is a crucial step. The literature and findings suggest that local governments and community organizations should foster greater community participation in reintegration programs, possibly through volunteer initiatives or partnerships with charities that support MCoSO. Involving community members can help shift attitudes by promoting shared responsibility for community safety and offering a pathway for MCoSO to regain a sense of belonging. This is especially important in overcoming the negative stereotypes that exacerbate marginalization.

The presence of MCoSO in residential neighborhoods evoked varied community concerns and fears for the public in the livelihood theme, primarily centered around the safety of children and the overall sense of security among residents. The economic impact of housing MCoSO was another significant concern. The potential decline in property values and negative effects on local businesses prompted residents to contemplate relocation. This economic impact highlights the need for balanced policies that address both community safety and the reintegration of offenders.

However, this review also highlighted instances of community mobilization and support for MCoSO. In some communities, there was a recognition of the importance of rehabilitation and providing second chances. Informal support networks and community engagement initiatives were examples of efforts to foster reintegration and promote understanding between residents and offenders. Neighborhood meetings and discussions facilitated by local authorities were seen as beneficial for the public in addressing concerns and promoting mutual understanding. Efforts at community engagement and support for MCoSO are supported by literature advocating for restorative justice and rehabilitation. Programs that promote community involvement and support for offenders have been shown to reduce recidivism and facilitate successful reintegration (Maruna & LeBel, 2002). These initiatives highlight the importance of empathy, understanding, and support in the rehabilitation process.

Public opinion on the rehabilitation through interventions of MCoSO was sharply divided; pessimistic perspectives argued that MCoSO possesses inherent character defects that predispose them to reoffend. This view was often fueled by the belief that reoffending rates for MCoSO were exceedingly high, although this notion is widely debated and often overstated (Stafford & Vandiver, 2017). Conversely, optimistic perspectives emphasize the human capacity for change and the potential benefits of rehabilitation programs. The divide in perspectives on the rehabilitation of sex offenders mirrors broader debates in the wider literature. The pessimistic view, which doubts the potential for rehabilitation and emphasizes high reoffending rates, often stems from historical perspectives on the inherent immutability of criminal behavior (Hanson & Morton-Bourgon, 2019). However, empirical evidence suggests that with appropriate interventions, the risk of reoffending can be significantly reduced (Hanson et al., 2014).

The effectiveness of treatment and rehabilitation programs was another contentious issue for the public. While some expressed skepticism about their efficacy, others highlighted the necessity of such programs and called for increased investment in rehabilitation efforts. The argument for comprehensive rehabilitation was rooted in the belief that addressing the underlying causes of offending behavior was essential for reducing reoffending and promoting long-term societal safety.

### Study Limitations

This systematic review is not without its limitations. One of which is the reliance on qualitative studies, which often focus on specific contexts and small sample sizes, potentially limiting the generalizability of the findings. Moreover, including studies from different countries with varying legal systems and cultural views on sexual offending may have introduced inconsistencies in the results, as societal attitudes toward MCoSO can be shaped by a range of cultural, religious, and political factors. This variation highlights the importance of contextual factors in shaping public opinion, meaning that findings from one country may not fully translate to another. Additionally, the diversity of participant samples within the included studies is a critical consideration. Since most of the research focused on male offenders in Western contexts, the findings may not be generalizable to other demographic groups. The perspectives of racial and ethnic minorities, for example, are underrepresented, despite evidence suggesting that public attitudes toward sexual offenders may vary significantly across different cultural and racial groups (Klein, 2015). These groups may experience different levels of stigma and marginalization based on intersecting factors such as race, socioeconomic status, and gender. Moreover, while much of the literature examines overall attitudes toward MCoSO, there is a significant gap in research that specifically addresses the reintegration process and the impact of interventions aimed at shifting public perceptions. Despite these challenges, the review remains highly valuable, offering a thorough synthesis of existing research, highlighting critical gaps in the literature, and providing a foundation for future studies to explore more detailed aspects of public attitudes toward MCoSO and their reintegration.

In light of the findings and limitations of this review, future studies should prioritize addressing the gaps in understanding public attitudes toward MCoSO, particularly in under-researched areas. First, there is a clear need for research that includes more diverse participant samples, specifically exploring how racial, cultural, and gender differences influence public perceptions of sexual offenders and their reintegration. Such studies should focus on non-Western contexts and marginalized communities, where intersecting factors such as race, age, ethnicity, and socioeconomic status may shape public attitudes in unique ways. In addition, future research should investigate the reintegration process from a longitudinal perspective, assessing the long-term impact of public education campaigns, community involvement initiatives, and enhanced support systems, such as CoSA.

Further attention should be given to understanding the effectiveness of interventions aimed at shifting public perceptions and reducing stigma, particularly in areas where misconceptions about sexual offending persist. Finally, more studies should examine the reintegration experiences of female and non-binary offenders, as current research overwhelmingly focuses on male offenders. By addressing these gaps, future studies can contribute to the development of more informed, effective policies that balance public safety with the potential for offender rehabilitation and successful reintegration into society.

## Conclusion

This systematic review has explored the complex and often conflicted public views on the reintegration of MCoSO into the community. The findings highlight the significant challenges posed by societal stigma, misinformation, and the tension between public safety and rehabilitation. Despite these challenges, there is a path forward through thoughtful policy-making that balances the need for community safety with the potential for offender rehabilitation. The review highlights the necessity of public education to combat myths, the importance of community support systems, and the need for a legal framework that is both just and effective. By focusing on rehabilitation and reintegration rather than solely on punishment, society can better support the long-term safety and well-being of all its members, including both potential victims and offenders. As attitudes continue to evolve, ongoing research and dialogue will be crucial in shaping policies that are humane, evidence-based, and capable of reducing recidivism. Ultimately, the goal should be to foster a society that supports rehabilitation while also safeguarding the rights and safety of the community.

**Table:** Summary of Critical Findings.

**Public Stigma and Misconceptions:** The review highlights that public views on MCoSO are predominantly negative, heavily influenced by media portrayals and widespread misconceptions about the risk of reoffending. This stigma leads to the public supporting stringent monitoring, restrictions, and punitive measures, which can hinder the reintegration of offenders and perpetuate a cycle of social isolation.
**Tension Between Community Safety and Rehabilitation:** There is a significant tension between the public’s desire for community safety and the potential for rehabilitation of MCoSO. While some members of the public advocate for supportive programs that facilitate reintegration, the prevailing view tends to favor punitive approaches, such as sex offender registries and strict supervision, often due to a lack of trust in rehabilitation efforts.
**Impact on Housing and Community Acceptance:** The review identifies that the presence of MCoSO in residential neighborhoods creates significant fear and discomfort among residents, leading to social ostracism and economic impacts, such as declining property values. This fear is exacerbated by the lack of accurate information and the public’s demand for transparency about offenders’ pasts, further complicating their reintegration into society.

**Table:** Implications for Practice, Policy, and Research.

**Public Education Campaigns:** Implement educational programs to address misconceptions and reduce stigma associated with MCoSO. These campaigns should focus on providing factual information about recidivism rates, the effectiveness of rehabilitation programs, the reality of the restrictions, the scope of the possible surveillance of registrants, and the benefits of supportive reintegration.
**Encouraging Community Involvement in Reintegration Efforts:** Community involvement is essential in the reintegration process. Local governments should encourage community members to participate in reintegration efforts, possibly through volunteer programs or partnerships with charity organizations that work with MCoSO. This can help foster a sense of shared responsibility for community safety and offender rehabilitation.
**Enhanced Support Systems for Reintegration:** Providing structured support systems for MCoSO including access to mental health services, substance abuse treatment, housing, and employment support. Policies should encourage the development of community-based support networks, such as Circles of Support and Accountability (CoSA), which have shown effectiveness in reducing recidivism and promoting positive outcomes for offenders.

## Supplemental Material

sj-docx-1-tva-10.1177_15248380251325816 – Supplemental material for A systematic review of public views on the reintegration of men convicted of a sexual offense into the community Supplemental material, sj-docx-1-tva-10.1177_15248380251325816 for A systematic review of public views on the reintegration of men convicted of a sexual offense into the community by Emma Tuschick, Shiri Portnoy, Nikki Carthy, Laura Gair, Simon Hackett and Nadia Wager in Trauma, Violence, & Abuse
