# Supplementary material for: A systematic review of public views on the reintegration of men convicted of a sexual offense into the community
Source: Trauma Violence Abuse. 2025 Mar 18;27(3):921–33. doi: 10.1177/15248380251325816 (PMC13287497; doi:10.1177/15248380251325816)
Supplement: sj-docx-1-tva-10.1177_15248380251325816 – Supplemental material for A systematic review of public views on the reintegration of men convicted of a sexual offense into the community [file sj-docx-1-tva-10.1177_15248380251325816.docx]

**Supplementary File 1. Search String**

| **P**opulation  AND | The general public | “public” OR “people” OR “community” OR “student*” OR “laypeople” OR “public opinion” |
| --- | --- | --- |
| **E**xposure AND | Reintegration of sexual offenders | “sexual offenders” OR “sex offenders” OR “rapists” OR “child sexual abusers” OR “paedophiles” OR “pedophiles” OR “child molester” OR “sex abuser” OR “sexual abuser” OR “online predator” OR “internet-facilitated sexual offenders” OR “CSEM offenders” OR “incest offender”  “reintegration” OR “re-entry” OR “reentry” OR “community” OR “transition” OR “resettlement”  “employment” OR “job” OR “work” OR “housing” OR “accommodation” OR “approved premises” OR “settlement” OR “education” OR “training” OR “community notification” OR “registry” OR “disclosure” OR “conditions” |
| **O**utcome | Views and opinions | “attitudes” OR “perceptions” OR “view*” OR “myths” OR “opinion” OR “fear” OR “negative” OR “positive” OR “apprehension” OR “beliefs” OR “factors” OR “problem” OR “thoughts” OR “support” OR “stereotype” |
